# Ectopic lipid metabolism in anterior pituitary dysfunction

**DOI:** 10.3389/fendo.2023.1075776

**Published:** 2023-02-13

**Authors:** Clemens Baumgartner, Martin Krššák, Greisa Vila, Michael Krebs, Peter Wolf

**Affiliations:** Department of Internal Medicine III, Division of Endocrinology and Metabolism, Medical University of Vienna, Vienna, Austria

**Keywords:** ectopic fat, HPA - hypothalamic-pituitary-adrenal, growth hormone, hypogonadism, thyroid hormone, NAFLD, cardiac steatosis

## Abstract

Over the past decades, adapted lifestyle and dietary habits in industrialized countries have led to a progress of obesity and associated metabolic disorders. Concomitant insulin resistance and derangements in lipid metabolism foster the deposition of excess lipids in organs and tissues with limited capacity of physiologic lipid storage. In organs pivotal for systemic metabolic homeostasis, this ectopic lipid content disturbs metabolic action, thereby promotes the progression of metabolic disease, and inherits a risk for cardiometabolic complications. Pituitary hormone syndromes are commonly associated with metabolic diseases. However, the impact on subcutaneous, visceral, and ectopic fat stores between disorders and their underlying hormonal axes is rather different, and the underlying pathophysiological pathways remain largely unknown. Pituitary disorders might influence ectopic lipid deposition indirectly by modulating lipid metabolism and insulin sensitivity, but also directly by organ specific hormonal effects on energy metabolism. In this review, we aim to I) provide information about the impact of pituitary disorders on ectopic fat stores, II) and to present up-to-date knowledge on potential pathophysiological mechanisms of hormone action in ectopic lipid metabolism.

## Introduction

Under physiologic conditions, white adipose tissue (WAT) inherits an essential role as a repository of energy. Uptake and processing of excessive nutrients and suppression of lipolysis enable energy-storage *via* accumulation of triglycerides (TG), ready for mobilization if needed. In state of overnutrition, WAT meets its protective purpose as metabolic sink for potentially harmful nutrient oversupply by continuous uptake and, concomitantly, progressive WAT expansion (1). However, the individual storage capacity is limited, wherefore WAT subsequently fails to expand in a state of chronically positive energy balance (1). By exceeding the individual fat threshold (2), TG further accumulate at ectopic sites other than WAT, resulting in an unfavorable increase of visceral fat, as well as ectopic lipid accumulation in insulin dependent organs (3).

Accumulation of visceral and ectopic fat is commonly related to impaired metabolic and cardiovascular health (4, 5). Organs affected by lipid accumulation include liver, myocardium, skeletal muscle, and pancreas, in which ectopic steatosis provokes function-impairing effects and parenchymal damage. When ectopic fat mass exceeds the organ specific oxidative capacity, this results in lipotoxicity and promotes local insulin resistance (IR), but also local organ damage and parenchymal dysfunction (6). Considered lipotoxic mechanisms are generation of reactive oxygen species, inflammation, and lipid-induced apoptosis, determined by lipotoxic metabolites of free fatty acids (FFA), such as diacylglycerols and ceramides (7).

Ectopic lipid content in organs important for whole body energy metabolism is crucial for cardiometabolic risk and its systemic complications (8). Of note, ectopic TG stores appear to be rather flexible and largely depend on circulating concentrations of substrates, including glucose, insulin and FFA (9, 10).

**Figure 1:**
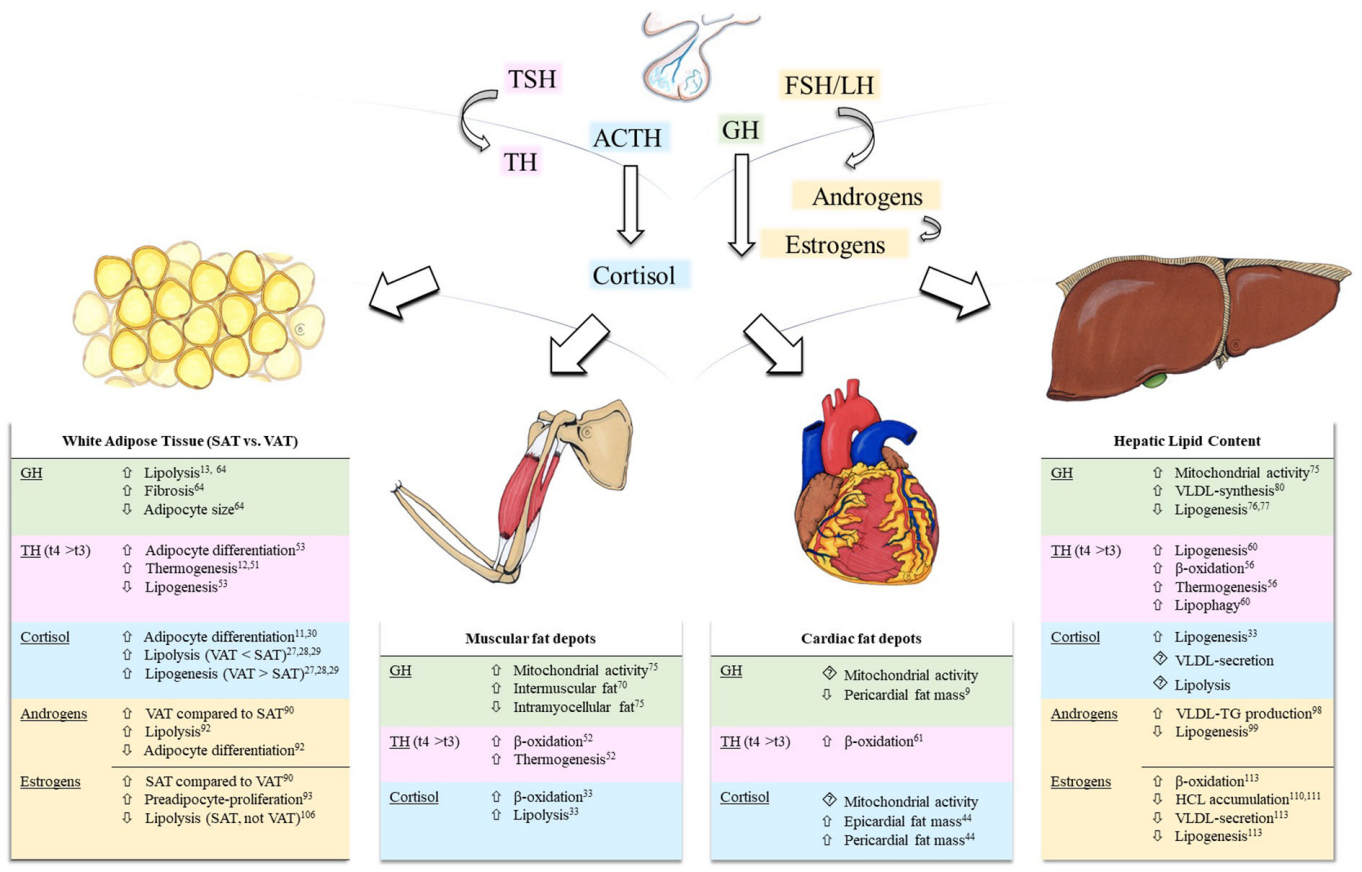
Effects of pituitary hormone axes on white adipose tissue and ectopic lipid content. Depicted pituitary axes include the thyroid-axis (*light pink*), growth hormone-axis (*green*), hypothalamic-pituitary-adrenal axis (*blue*), and the hypothalamic-pituitary-gonadal axis (*yellow*). Modulations of ectopic lipid content are summarized in tables for each effector (GH, TH, Cortisol, Androgens, and Estrogens) in skeletal muscle, liver, and heart. Reference numbers are attached as superscript. Background colors correspond to the depicted hormonal axes. Hypothalamic hormones, negative feedback loops, and, due to scarcity of data, prolactin are not shown. ACTH, adrenocorticotropic hormone; FSH, follicle-stimulating hormone; GH, growth hormone; HCL, hepatic lipid content; LH, luteinizing hormone; SAT, subcutaneous adipose tissue; TG, triglycerides; TH, thyroid hormones; TSH, thyroid-stimulating hormone; t4, thyroxine, t3, triiodothyronine; VAT, visceral adipose tissue; VLDL, very-low density lipoprotein.

Beside metabolic conditions that favor an increase in ectopic lipids, such as obesity and diabetes mellitus, hormones controlled by the anterior pituitary gland are also frequently reported to modulate lipid storage (**Figure 1**). The anterior pituitary sets the pulse for peripheral secretion of cortisol, thyroid hormones, and sex hormones, and also releases growth hormone (GH) and prolactin into circulation. Alongside other well-known properties, these effectors are tightly related to alterations in lipid metabolism (11–15).

The underlying pathophysiological mechanisms for ectopic TG deposition and its clinical relevance is best described for the insulin resistant state. As pituitary disorders are commonly associated with metabolic diseases, TG accumulation in non-adipose tissue organs might play a relevant role in these conditions (16). Of note, ectopic lipid metabolism is tightly connected to glucose homeostasis and changes in ectopic fat mass are usually related to changes in insulin resistance (17). Previous reviews have extensively summarized the impact of anterior pituitary hormones on glucose metabolism and dyslipidemia (18–22). A detailed discussion of those topics therefore is beyond the scope of this review. Nevertheless, the relationship between hormonal excess or deficiency, circulating substrate concentrations and the impact on ectopic TG storage is complex, due to direct, organ specific effects of pituitary hormones. Here we give an overview on the current knowledge of ectopic lipid metabolism in pituitary hormone syndromes categorized by their main metabolically active hormones.

## Cortisol

The hypothalamus-pituitary-adrenal axis is a major player in the regulation of energy metabolism. In Cushing’s disease, the metabolic syndrome is highly prevalent (23, 24). Cortisol exerts insulin-antagonistic effects by inhibiting insulin secretion, stimulating glucagon secretion, and disrupting insulin signaling. In addition, enhanced hepatic gluconeogenesis and glycogenolysis in combination with increased FFA concentrations following adipose tissue lipolysis contributes to IR (16).

The glucocorticoid receptor is known to stimulate and accelerate adipogenesis (25, 26). Cortisol stimulates lipolysis in adipose tissue directly and indirectly by enhancing the sensitivity to catecholamines (27). However, the lipolytic activity in abdominal visceral fat might be lower compared to other tissues due to local differences in glucocorticoid receptor expression (28, 29). Moreover, cortisol both stimulates and inhibits lipogenesis, which depends on the extent of hypercortisolism, the duration of glucocorticoid exposure and the presence of insulin (11, 30). Crucial factors regulating these effects include the enzyme 11β-hydroxysteroid dehydrogenase I (11β-HSD1), which locally activates cortisol by conversion from cortisone, and whose activity might change following the prolonged exposure to high doses of glucocorticoids in Cushing’s syndrome (31). Furthermore, an important role of the AMP-activated protein kinase (AMPK) has been discussed, which is a key metabolic regulator of cellular energy status. A downregulation of AMPK in visceral adipose tissue was observed in patients with Cushing’s syndrome, which inversely correlated with the degree of hypercortisolism (32). These cortisol mediated changes in lipid metabolism might explain the typical phenotype of adipose tissue distribution in patients with Cushing’s disease, characterized by an increase in visceral obesity and a loss of peripheral subcutaneous fat depots (33).

Interestingly, despite the huge amount of evidence on the effects of cortisol on impaired glucose homeostasis, only limited knowledge exists on ectopic TG accumulation in non-adipose tissue organs in a state of chronic hypercortisolism. On the background of IR and increased concentrations of FFA in patients with Cushing’s disease, one might assume that ectopic TG mass in skeletal muscle is higher. However, studies in humans confirming these effects are rare. Moderate exogenous hypercortisolemia by substitution with hydrocortisone in combination with strict physical inactivity almost doubled the intramuscular lipid content in healthy volunteers (34). Moreover, higher diurnal salivary cortisol levels were associated with higher intramuscular fat mass in healthy volunteers (35). In patients with biochemically cured Cushing’s syndrome, intramuscular fat was higher compared to a matched control group and was negatively associated with the performance on functional tests (36).

In the liver, a relation between hypercortisolism and of ectopic intrahepatic TG accumulation, also termed non-alcoholic fatty liver disease (NAFLD), was suggested (37). Most metabolic pathways modulating hepatic lipid content (HCL) are influenced by cortisol directly or indirectly by its effects on IR. Glucocorticoids regulate several genes involved in *de-novo* lipogenesis (DNL). In addition, elevated concentrations of glucose and insulin together with increased FFA flux from adipose tissue into the liver stimulate TG synthesis (38). Moreover, cortisol modulates beta oxidation and secretion of very-low density lipoprotein (VLDL) (18), suggesting a net retention of fat within the liver.

Elevated intrahepatic fat content is associated with inadequate suppression of cortisol following the overnight administration of dexamethasone (39). Furthermore, an increased prevalence of NAFLD in patients with Cushing’s syndrome was reported (40). However, up to now no studies using proton magnetic resonance spectroscopy, which is the gold standard method to non-invasively investigate hepatic fat *in-vivo* (41), have been published in patients with active Cushing’s disease.

With regards to the heart, studies using cardiac magnetic resonance imaging reported an increase in left ventricular mass and a modest reduction in systolic function in patients with active Cushing’s disease, although the prevalence of overt cardiomyopathy was lower than previously reported in ultrasound based investigations (42, 43). The observed changes in cardiac function and morphology are both potentially reversible after successful treatment of hypercortisolism (43). The increase in ventricular mass surprisingly contrasts with general skeletal muscle atrophy related to protein wasting, which is typically present in patients with Cushing’s disease. On the background of visceral obesity, IR, and dyslipidemia, it could be assumed that cardiac steatosis might play an important role in the development of myocardial hypertrophy. However, no differences in intramyocardial TG content were found neither in patients with Cushing’s syndrome compared to controls, nor in patients with Cushing’s syndrome before and after normalization of hypercortisolism. This is probably explained by higher rates of beta oxidation within the myocardium stimulated by cortisol excess, which might prevent the heart from the development of cardiac steatosis (44). On the other hand, an important increase in epicardial fat mass was observed compared to controls, which decreased following biochemical disease remission (44, 45). Epicardial fat is a well-known mediator of inflammation, microvascular dysfunction, and fibrosis (46). By directly surrounding the myocardium, it might exert paracrine effects by adipocytokine secretion (46). Epicardial fat might therefore play an important role in the development of heart disease in hypercortisolism. Biochemical disease remission decreased epicardial fat after a median follow up of 9 months, which highlights the important impact of cortisol.

On the contrary, in patients suffering from adrenal insufficiency even a small oversupply of daily glucocorticoid substitution therapy was associated with an adverse cardiometabolic risk profile, characterized by an increase in visceral adipose tissue, higher fasting glucose values and hypertension (47). However, in a cohort of patients with state-of-the-art hormone replacement therapy, no differences in visceral WAT mass could be found compared to a healthy control group in a cross sectional study (48). Ectopic fat accumulation in the liver and myocardium was similar in patients with adrenal insufficiency compared to a control cohort with physiological hypothalamic-pituitary-adrenal axis signaling (49). In addition, no difference in visceral and ectopic lipid distribution was observed, when patients with a daily dose of > 20 mg and ≤ 20 mg were compared (49).

## Thyroid hormone

Thyroxine (T4), a prohormone, which is converted to triiodothyronine (T3) to acquire full biological activity, is the major secretory product of the thyroid gland and is controlled by the thyroid-stimulating hormone (TSH) secreted from the anterior pituitary gland (50). Primary hypothyroidism constitutes one of the most common endocrine diseases and is linked to a variety of changes in lipid metabolism.

Hypothyroidism has been associated with visceral and ectopic fat accumulation. In WAT, thyroid hormones regulate adipogenesis and the proliferation and differentiation of adipocytes. Furthermore, T3 regulates thermogenesis and increases resting energy expenditure, probably by stimulating a trans-differentiation from white to beige adipocytes (12, 51). Similar effects have also been described in skeletal muscle, in which T3 promoted thermogenesis by mitochondrial energy uncoupling (52). Furthermore, thyroid hormones regulate gene expression for lipogenesis and lipolysis in white adipose tissue, and thermogenesis in brown adipose tissue (53).

Regarding ectopic fat stores, hypothyroidism has been linked with TG accumulation in the liver and the myocardium, but not in skeletal muscle. In the general population, there is an inverse association between T4 and intrahepatic fat accumulation (54). This association can be observed in both subclinical and overt hypothyroidism and is independent of differences in the BMI (55). Organ specific activation of the thyroid hormone receptor in the liver prevents the development of hepatic steatosis in animal studies (56) and is currently evaluated as therapeutic agent in the context of NAFLD (57). However, we previously failed to demonstrate a reduction in HCL following the treatment of overt hypothyroidism (58, 59), which might be explained by the short period of hypothyroidism before study inclusion. The effects of thyroid hormones on hepatic lipid metabolism are complex. *De-novo* intrahepatic lipogenesis is stimulated by thyroid hormones because of increased FFA uptake, but also lipogenic gene expression. However, there are also catabolic effects of thyroid hormones, including lipolysis, TG autophagy and mitochondrial beta oxidation (60). Therefore, FFA metabolism occurs at a higher rate than fatty acid synthesis.

In the myocardium, ectopic lipid stores decrease following the initiation of a treatment by levothyroxine independent of changes in body weight in patients with severe primary hypothyroidism (58). This might be explained by changes in myocardial mitochondrial lipid oxidation (61), or by alterations in FFA uptake within the heart (62). The reduction in intramyocardial fat mass following treatment of hypothyroidism was associated with significant improvements in systolic left ventricular heart function (58).

## Growth hormone

In the context of ectopic fat disposal, GH and its exceptional properties on lipid metabolism are of particular interest. Redistribution and enhanced utilization of lipids are frequently reported in acromegaly, a state of increased GH activity (13). On the other hand, overall and visceral adiposity are characteristics of GH deficiency (GHD) (63). The investigation of these endogenous models of GH excess and insufficiency might therefore help to determine the clinical relevance of antisteatotic GH action and its benefits on lipid profile.

Human GH is a 22kDa hormone produced and secreted by somatotropic cells of the anterior pituitary gland. Underlying a pulsed secretion with higher peaks at night-time, circulating GH stimulates the production of insulin-like growth factor 1 (IGF1), predominantly in the liver, which in turn executes various effects in diverse organs and tissues. With IGF1 inhibiting GH secretion, the hormonal axis underlies a self-controlling feedback mechanism (13, 64, 65).

Under physiologic conditions, GH is a main regulator of energy metabolism during stress and famine, where it is considered to preserve proteins and sugars by shifting catabolism to the exploitation of lipids. In this regard, GH induces lipolysis in WAT, promoting the release of FFA into circulation, and increases lipid oxidation (13). Therefore, GH is considered to improve body composition by degradation of fat stores, while concomitantly increasing lean body mass (66). On the other hand, GH indirectly stimulates adipogenesis by IGF1, which impacts adipocyte proliferation and differentiation (67). IGF1 is of major significance in the differentiation of pre-adipocytes into adipocytes and stimulates the proliferation of adipocyte precursor cells (68).

Acromegaly states a condition in which patients suffer from constantly high GH concentrations, originating from a somatotropic adenoma of the pituitary gland in almost all cases. Patients with acromegaly show a disease specific phenotype of lipid distribution (69). Visceral and subcutaneous WAT are about 70% and 80% lower in patients with active acromegaly compared to controls and adipose tissue distribution is significantly associated with disease severity (70, 71). Following pituitary surgery and disease remission, WAT increases substantially and trends to normalize compared to studied cohorts of healthy controls (71, 72). However, following insulin-antagonizing effects of GH, acromegalic patients exhibit a unique form of IR despite a low body fat content (73), which is pathophysiologically connected to the increase in WAT lipolysis (13). Both IR and the reduction in fat mass decline after treatment (72). Furthermore, acromegaly also represents a unique condition of very low ectopic fat mass despite severe IR. In active acromegaly, a low amount of ectopic fat mass has been reported in the liver and in skeletal muscle, concomitantly with increased mitochondrial activity (9, 74). However, hepatic ATP-turnover indicating mitochondrial activity showed to be rather modest and might not fully explain the very low amounts of HCL, wherefore it is likely that GH mediates additional antisteatotic effects within the liver. In addition to the increase in mitochondrial activity, other potential GH-induced mechanisms on hepatic lipid storage might include the inhibition of DNL and increased hepatic VLDL export (75–79). To date, GH-mediated effects on hepatic DNL have been studied in mouse models, in which liver specific GH-receptor knockdown led to an increase in DNL favoring hepatic steatosis (75). Suppressive effects of GH on DNL by down-regulation of carbohydrate responsive element-binding protein and fatty acid synthase were also reported in cultured human HepG2 hepatocytes of an *in-vitro* steatosis model (80). However, *in-vivo* studies confirming these effects in human subjects are missing. On the other hand, first investigations of GH influencing hepatic VLDL secretion in human subjects have already been made: Hepatic lipid oversupply is compensated by an increase in TG export *via* VLDL particles. However, as HCL rises above 10%, VLDL secretion cannot be further intensified, wherefore a net retention of lipids leads to a progression of NAFLD (81). A hormone-mediated activation of VLDL secretion is not unlikely: Recently, our study group proposed a Leptin-mediated increase of VLDL secretion *via* a brain-vagus-liver axis to protect against elevated HCL (82). Studies investigating the impact of GH on VLDL secretion are rather conflicting, although their methodological approaches differed substantially. Following a 3-month period of GH replacement therapy, a significant increase of VLDL secretion was observed in patients with GHD (77). On the contrary, 8 days of GH administration did not show any changes in VLDL kinetics (78).

Regarding skeletal muscle, not intra-, but intermuscular fat was elevated in acromegalic subjects observed by Freda et al., which hypothesized a connection to muscular IR present in acromegaly (70). This lipid redistribution was lately proposed to be termed an acromegaly-specific lipodystrophy (83). Considering cardiac fat depots, no differences in intramyocardial lipid content between an acromegaly cohort and healthy controls could be observed. However, pericardial fat mass increased after treatment of acromegaly by transsphenoidal pituitary surgery (9).

Contrary to acromegalic fat distribution, GHD-patients inherit IR alongside an elevated visceral and ectopic fat mass in combination with a concomitantly reduced lean body mass (84). Compared to healthy controls, the predominant increase of ectopic fat in GH-deficient individuals was seen in the liver, whereas differences in ectopic lipid deposition in skeletal muscle did not reach statistical significance (85). In GH-deficient patients, as well as in abdominally obese volunteers with IGF1 levels in the lower normal range, low-dose substitution of GH resulted in an improvement of body composition and in a reduction of HCL (63, 66). Moreover, insulin sensitivity tended to rise in GHD patients after low-dose GH substitution (86), indicating the diverse entities of IR in acromegaly and GHD. In another study, GH replacement was followed by an increase in lipid oxidation (87).

## Sex hormones

The hypothalamus-pituitary-gonadal axis is well-known for its impact on body composition, glucose and lipid metabolism (14). Adipose tissue is a crucial target for sex hormones in humans. Especially androgens are characterized by a sexual dimorphism, with a tendency towards an increased accumulation of visceral fat in men and a higher proportion of subcutaneous and peripheral fat in women (88, 89). Sex hormones have a well-known impact on adipocytes in a sex specific manner. The physiological and pathophysiological role of androgens and estrogens on adipose tissue function, adipocyte proliferation and differentiation has been extensively reviewed previously (90, 91). However, evidence on the direct impact of sex hormones on ectopic fat is relatively scarce.

In men, a reduction in concentrations of circulating testosterone is associated with obesity, IR, and hypertension. Moreover, hypogonadism in men suffering from prostate cancer treated with GnRH agonists is associated with a rise in fat mass (92). Considering ectopic fat stores, retrospective studies reported an increased prevalence of hypogonadism in patients with NAFLD (93). Replacement of testosterone improved insulin sensitivity and reduced fat mass in hypogonadal men with type 2 diabetes mellitus (T2DM) (94), but did not have any effects on endogenous glucose production or glucose disposal rates following medically induced short-term hypogonadism (95). Furthermore, lipid oxidation is lower in an early hypogonadal state and high, but physiological doses of testosterone increased VLDL secretion (96). Androgen treatment also lowered DNL in patients with AIDS-wasting-syndrome and borderline low serum testosterone (97). However, until now, no effects on HCL were observed following the initiation of testosterone replacement therapy in patients with T2DM, as well as in elderly, hypogonadal men with abdominal obesity (98, 99). Furthermore, short term hypogonadism by biochemical castration had no effects on intramyocellular lipids in healthy volunteers (100). Of note, the effects of testosterone replacement on changes in body composition and ectopic fat accumulation might correlate with circulating estrogen concentrations and aromatase activity in men (101, 102).

In women, the risk for the development of NAFLD increases with age and premenopausal women appear to be protected from ectopic fat accumulation (103). Despite a lower skeletal muscle mass compared to men, premenopausal women show a higher energy-storage capacity in subcutaneous WAT (104). These changes in regional WAT distribution might be explained by effects of estrogen, which attenuates lipolysis in subcutaneous, but not in visceral adipose tissue (105). Regarding different regions of ectopic fat, the absolute amount of intramyocellular fat in the skeletal muscle appears to be higher in women compared to men. However, the relative amount of potentially toxic intermediates of lipid metabolism, i.e. diacylglycerol and ceramide, was lower in females (106). Furthermore, animal models show an increase in ectopic lipid content in skeletal muscle following ovariectomy in rodents (107).

Within the liver, pre-menopausal women might be protected against hepatic steatosis, since NAFLD prevalence increases with age (108). Moreover, women treated with the estrogen receptor antagonist tamoxifen have a higher risk to develop NAFLD (109). In ovariectomized female mice estrogen replacement attenuates hepatic fat accumulation following high fat diet (110). Possible mechanisms might include an acceleration of hepatic VLDL secretion, inhibition of DNL and increased beta oxidation (111). Of note, when matched for HCL, FFA oxidation and DNL is higher in men compared to pre-menopausal women, which might explain the pro-atherogenic risk profile (112). In addition to sex hormones, the sex dependent pattern of GH secretion pulsatility is a major regulator of intrahepatic lipid metabolism (113).

## Prolactin

Hyperprolactinemia is associated with weight gain and insulin resistance and an increased prevalence of obesity has been reported in prolactinoma patients (15). Normalization of prolactin levels after treatment resulted in weight reduction in some studies (114), but not in others (115). Furthermore, treatment with dopamine agonists might have beneficial effects on body weight (116) and other metabolic parameters (117). In this regard, it is presumed that bromocriptine action resets an abnormally elevated hypothalamic drive for increased plasma glucose and lipids in IR by modulating circadian neuronal activities (118). Underlying pathophysiological mechanisms are unclear but might include changes in the dopaminergic tone (119), but also the presence of concomitant hypogonadisms might be of importance.

Studies investigating ectopic fat depots in prolactinoma patients are missing. In a report of a single patient NAFLD improvement following treatment with a dopamine agonist (120). On the contrary, cross-sectional retrospective studies in patients without pituitary disorders showed that low prolactin concentrations are associated with an increased risk for hepatic steatosis (121). Additionally, prolactin levels were lower in men and women with severe hepatic steatosis compared to patients with only mild hepatic steatosis (122).

## Future perspectives

In conclusion, our review presents current knowledge regarding the importance of anterior pituitary hormonal axes on ectopic lipid content. Disturbances of glucose and lipid homeostasis are frequently observed in pituitary hormone syndromes. Treatment of hormonal excess or deficiency has a profound impact on whole body energy metabolism and therefore also on changes in ectopic fat stores.

Additionally, since metabolic disorders are closely associated with ectopic lipid deposition, treatments reducing ectopic fat in organs pivotal for whole body metabolism might be of particular interest. Elucidating various hormonal effects on organ specific lipid metabolism might improve our understanding on the pathophysiological background of ectopic fat accumulation. This could be relevant to identify potential future drug targets for the development of novel, local antisteatotic therapies.

## Author contributions

All authors substantially contributed to the writing of the manuscript. All authors contributed to the article and approved the submitted version.
